# Exploring Paxlovid Efficacy in COVID-19 Patients with MAFLD: Insights from a Single-Center Prospective Cohort Study

**DOI:** 10.3390/v16010112

**Published:** 2024-01-12

**Authors:** Mykhailo Buchynskyi, Valentyn Oksenych, Iryna Kamyshna, Oleksandr Kamyshnyi

**Affiliations:** 1 Department of Microbiology, Virology, and Immunology, I. Horbachevsky Ternopil National Medical University, 46001 Ternopil, Ukraine; 2 Broegelmann Research Laboratory, Department of Clinical Science, University of Bergen, 5020 Bergen, Norway; 3 Department of Medical Rehabilitation, I. Horbachevsky Ternopil National Medical University, 46001 Ternopil, Ukraine

**Keywords:** COVID-19, MAFLD, Paxlovid, nirmatrelvir

## Abstract

This study investigates the intricate interplay between Metabolic-associated Fatty Liver Disease (MAFLD) and COVID-19, exploring the impact of MAFLD on disease severity, outcomes, and the efficacy of the antiviral agent Paxlovid (nirmatrelvir/ritonavir). MAFLD, affecting a quarter of the global population, emerges as a potential risk factor for severe COVID-19, yet the underlying pathophysiological mechanisms remain elusive. This study focuses on the clinical significance of Paxlovid, the first orally bioavailable antiviral agent granted Emergency Use Authorization in the United States. Notably, outcomes from phase II/III trials exhibit an 88% relative risk reduction in COVID-19-associated hospitalization or mortality among high-risk patients. Despite conflicting data on the association between MAFLD and COVID-19 severity, this research strives to bridge the gap by evaluating the effectiveness of Paxlovid in MAFLD patients with COVID-19, addressing the scarcity of relevant studies.

## 1. Introduction

In an endeavor to gain a more comprehensive understanding of COVID-19 and identify potential therapeutic interventions, the pandemic has given rise to scientific investigations that have unveiled novel insights into the intricate interplay between Metabolic-associated Fatty Liver Disease (MAFLD) and infection [1,2].

MAFLD, a prevalent cause of chronic liver disease, affects a quarter of the global population [3,4]. It is recognized as a sensitive and pivotal indicator of metabolic dysfunction [3].

Several studies posit that MAFLD constitutes a noteworthy risk factor for the acquisition of Severe Acute Respiratory Syndrome Coronavirus-2 (SARS-CoV-2) and subsequent hospitalization, independently of other components of the metabolic syndrome. Moreover, there is a potential association with heightened disease severity, prolonged hospitalization, and unfavorable outcomes [5,6]. Nevertheless, the pathophysiological mechanisms through which MAFLD exacerbates COVID-19 remain undisclosed. One proposed hypothesis suggests that MAFLD exacerbates the phenomenon of the “cytokine storm” through the hepatic release of pro-inflammatory cytokines [7]. Current research indicates that COVID-19 patients with coexisting MAFLD exhibit a distinct cytokine profile, characterized by elevated levels of interleukin (IL)-6, IL-8, IL-10, and C-X-C motif chemokine ligand 10 (CXCL10), all of which are implicated in a more severe clinical presentation [8,9,10].

Meta-analyses have postulated that the presence of MAFLD heightens the risk of severe progression of COVID-19 and augments the likelihood of patients requiring admission to intensive care units [1,6,11,12,13]. However, its impact on the development of critical COVID-19 or mortality remains equivocal [14].

Beyond the exploration of comorbid pathologies, the treatment of COVID-19 persists as a paramount focal point in contemporary research endeavors [15,16]. The administration of an intravenous (IV) drug within a clinical setting proves suboptimal for addressing COVID-19 during its early stages when the maximal benefits of antiviral interventions are most likely to be realized.

Nirmatrelvir, a potent and highly specific inhibitor targeting the main protease of SARS-CoV-2, in conjunction with ritonavir—a Cytochrome P450 3A4 (CYP3A4) inhibitor employed as a pharmacokinetic enhancer—constitutes an orally bioavailable antiviral agent (PAXLOVID^TM^; Pfizer Inc., Tokyo, Japan) and has attained Emergency Use Authorization (EUA) in the United States for the treatment of COVID-19 [17]. Outcomes from the phase II/III trials revealed an 88% relative risk reduction in COVID-19-associated hospitalization or mortality among high-risk patients with moderate COVID-19 who initiated nirmatrelvir/ritonavir treatment within ≤5 days of symptom onset [18,19,20].

While research on the nexus between MAFLD and COVID-19 is limited and often yields conflicting results, there exists a dearth of studies elucidating the efficacy of Paxlovid treatment in individuals with COVID-19 concomitant with MAFLD. Our investigation seeks to bridge this gap by scrutinizing the association between MAFLD and the severity of COVID-19, alongside other pertinent outcomes, among patients treated with Paxlovid and laboratory-confirmed COVID-19.

## 2. Materials and Methods

### 2.1. Study Design and Population

This monocentric study was conducted at the I. Horbachevsky Ternopil National Medical University (TNMU), Ukraine, and was part of the prospective cohort study that recruited patients with COVID-19 with the aim to investigate the potential role and impact of MAFLD on COVID-19 severity and outcomes.

Seventy-two adults who tested positive for SARS-CoV-2 and were subsequently admitted to the hospital during the timeframe spanning from October 2022 to May 2023 were encompassed in the study. Confirmation of SARS-CoV-2 infection was established through the real-time polymerase chain reaction (RT-PCR) method, utilizing nasopharyngeal swab samples.

The inclusion criteria were adult patients with COVID-19 requiring hospital admission and classified according to the National Institute of Health (NIH) guidelines in moderate, severe, or critical COVID-19 [21].

Exclusion criteria encompassed patients admitted to the Intensive Care Unit (ICU) within 24 h of hospitalization, those who succumbed within the initial 48 h, individuals on corticosteroids pre-enrollment, and those with bacterial infections at admission. Additionally, exclusion criteria comprised individuals with known chronic liver disease and cirrhosis, active malignancies, alcoholism, pregnancy, receipt of parenteral nutritive support, immunocompromised status, including HIV-positive patients, and patients in palliative care.

Upon admission, a comprehensive screening for components of metabolic syndrome was conducted among patients. Patients were subsequently diagnosed with MAFLD based on contemporary criteria, relying on the identification of steatosis through diverse modalities (such as imaging, blood biomarkers, or histology), concomitant with the presence of at least one of three criteria: overweight or obesity, type 2 diabetes mellitus, or discernible evidence of metabolic abnormalities [22,23]. The hepatic steatosis index (HIS; AUROC of 0.812; 95% CI 0.801–0.824) was calculated to determine the presence of hepatic steatosis [23].

Depending on the severity of the disease, according to the NIH classification, patients were classified into 3 subgroups—moderate (bilateral pneumonia with SpO_2_ ≥ 94% on room air), severe (dyspnea and/or tachypnea > 24/min and/or SpO_2_ < 94%), and critical COVID-19 (requiring intensive care unit care, criteria for ARDS, advanced respiratory support with HFNC, non-invasive or invasive mechanical ventilation) [21].

All patients received standard treatment in accordance with the national treatment protocol for COVID-19. This regimen encompassed symptomatic antipyretic therapy (utilizing paracetamol or ibuprofen), mucolytic agent and expectorant (Ambroxol), anticoagulant therapy (administered through low-molecular-weight heparins, such as enoxaparin at a dosage of 40 mg or 4000 IU anti-Xa), antimicrobial treatment for co-infections (comprising amoxicillin/clavulanate in addition to macrolides such as azithromycin or clarithromycin, or cephalosporins of the II–III generation alongside macrolides), corticosteroids (administered intravenously at a dose of 0.15 mg/kg of dexamethasone once daily, with a dose of 8–16 mg, for a duration of 7–10 days), and non-invasive oxygen support.

The primary outcome was the length of hospital stay (number of days spent by participants in the hospital from the day of admission up to the day of their discharge). The secondary outcomes included the SpO_2_ level after–before dynamics depending on Paxlovid treatment.

Finally, 33 patients with MAFLD and 39 without MAFLD were included in the study. Eleven patients from the MAFLD group and twelve patients from the non-MAFLD group were administered nirmatrelvir–ritonavir (Paxlovid) according to the Food and Drug Administration (FDA) recommendations [24].

The enlisted participants were not previously involved in any prior investigations, and each individual explicitly granted written informed consent. This study was approved by the I. Horbachevsky Ternopil National Medical University Ethics Committee (protocol No. 72).

### 2.2. Laboratory and Clinical Data

At the time of hospital admission, baseline patient characteristics, including comorbidities, baseline clinical status, and vital parameters were collected.

As part of the standard diagnostic procedure, routine laboratory tests were collected, including oxygen saturation, white blood cell count (WBC), absolute neutrophil and lymphocyte count (ANC and ALC, respectively), erythrocyte sedimentation rate (ESR), platelet count (Plt), hematocrit, international normalized ratio (INR), prothrombin time (PT), quick prothrombin time (QTP), activated partial thromboplastin time (APTT), fibrinogen, total bilirubin, alanine aminotransferase (ALT), aspartate aminotransferase (AST), serum creatinine, gamma-glutamyl transferase (GGT), total protein, albumin, alkaline phosphatase (ALP), C-reactive protein (CRP), and blood glucose.

Anthropometric measurements, including body mass index (BMI), were documented.

### 2.3. Statistical Analysis

The clinical characteristics, laboratory parameters, and demographic information underwent meticulous assessment, and their presentation was conducted through descriptive statistics, featuring frequencies and medians along with interquartile ranges. To compare the two independent groups, Fisher’s exact test and the Mann–Whitney U test were employed. For comparisons involving three or more groups, the Kruskal–Wallis test with Dunn’s multiple comparisons test was applied. In instances requiring a comparison between two related groups, the Wilcoxon signed-rank test was utilized. All statistical tests conducted were two-tailed, with statistical significance defined as a *p*-value less than 0.05. Spearman’s correlation was used with two continuous variables, the point-biserial correlation between binary and continuous data, and the Chi-square test between two binary data, summarized in a correlation matrix. ROC analysis was used to assess the quality of a binary logistic regression model. Comparing time to hospital discharge between Paxlovid and standard therapy groups was evaluated using the Kaplan–Meier method and hazard ratios (HR) with 95% confidence intervals (95% CI) and *p*-values that were calculated via the log-rank test. Risk factors associated with COVID-19 severity, the need for oxygen supply, and factors to predict the Paxlovid therapy were investigated using a univariate and subsequently multivariable logistic regression analysis. The strength of association was expressed as an odds ratio (OR) and its corresponding 95% CI. Statistical analyses were performed using GraphPad Prism Software version 8.4.3 (San Diego, CA, USA), IBM SPSS Statistics 25, and Jamovi 2.4.11.

## 3. Results

### 3.1. Baseline Patients’ Characteristics

Of the 72 included patients, 33 patients were classified into the MAFLD group (63.6% males; the median age of 66, IQR 50–72) and 39 were classified into the non-MAFLD group (54.4% males; the median age of 65 IQR 41–72). There were no differences in demographics peripheral oxygen saturation, the need for oxygen supply, COVID-19 severity, and some comorbidities (arterial hypertension, chronic obstructive pulmonary disease (COPD), coronary heart disease, community-acquired pneumonia), as presented in Table 1. The median time interval from disease onset to hospital admission was similar between the groups (11, IQR 9–13 vs. 10 IQR 8–12, *p* = 0.082). There was, however, the MAFLD group had a statistically significant difference in BMI (30.8 kg/m^2^, IQR 28.42–33.5 vs. 24 kg/m^2^, IQR 22.4–25.35, *p* < 0.001), presence of T2DM (14, 42.4% vs. 2, 6.6%), *p* < 0.001) and obesity (18, 54.5% vs. 0).

Laboratory findings at admission are shown in Table 2. Patients in the MAFLD group had a higher aspartate aminotransferase level (27 mmol/L, IQR 21.4–43.6 vs. 22.9 mmol/L, IQR 16.6–27.7, *p* = 0.024), creatinine level (104 mmol/L, IQR 21.4–43.6 vs. 90 mmol/L, IQR 77–104, *p* = 0.015), gamma-glutamyl transferase level (60 U/L, IQR 35.5–87 vs. 36 U/L, IQR 23–66, *p* = 0.017), total protein level (70.9 g/L, IQR 64.6–76.25 vs. 61.1 g/L, IQR 61.2–70.4, *p* = 0.016), C-reactive protein level (12 mg/L, IQR 6–24 vs. 6 mg/L, IQR 6–12, *p* = 0.006) on admission, a higher hematocrit level (37.8%, IQR 31.97–45.95 vs. 34.5%, IQR 31.29–38.95, *p* = 0.028), gamma-glutamyl transferase level (67 U/L, IQR 41–93.5 vs. 43 U/L, IQR 30–65, *p* = 0.012), and lower total protein level (63.4 g/L, IQR 59.3–67.8 vs. 67.6 g/L, IQR 61.9–72.2, *p* = 0.019) on discharge.

We employed multinomial logistic regression analysis to discern factors influencing the severity of the COVID-19 disease (Table 3) and the simple logistic regression for the need for oxygen supply (Table 4). The first predictive model was developed conditioning on SpO_2,_ % (admission); lymphocytes, % (admission); QPT, % (admission); and albumin, g/L (admission).

The ensuing regression model demonstrates statistical significance (*p* < 0.001). The Nagelkerke R² value of 0.789 signifies a robust association between the predictors and the observed severity of COVID-19. Furthermore, this model achieves an accuracy of 86,1%, accurately predicting the outcomes.

Comparing moderate and severe COVID-19 regression model presented 1.123 times decreased SpO_2_ (admission) odds ratio (*p* < 0.001), 0.051 times decreased lymphocytes (admission) odds ratio (*p* = 0.160), 0.004 times decreased QPT (admission) odds ratio (*p* = 0.882), 0.050 times albumin (admission) odds ratio (*p* = 0.237).

Comparing moderate and critical COVID-19 models predict 2.382 times decreased SpO_2_ (admission) odds ratio (*p* = 0.092), 0.079 times decreased lymphocytes (admission) odds ratio (*p* = 0.733), 0.206 times decreased QPT (admission) odds ratio (*p* = 0.378), and 0.082 times albumin (admission) odds ratio (*p* = 0.808).

The second predictive model was developed conditioning on SpO_2_ (admission); leukocytes 10^9^/L (admission); hematocrit, % (admission); and creatinine, mmol/L (admission).

The resulting regression model is statistically significant (*p* < 0.001). Nagelkerke R² 0.811 indicates a strong relationship between predictors and observed the need for oxygen supply. The model achieves a high predictive accuracy, with 91.5% of predictions correctly classified.

This model showed 1.245 times decreased SpO_2_ (admission) odds ratio (*p* = 0.001), 0.261 times increased leukocytes (admission) odds ratio (*p* = 0.225), 0.067 times increased hematocrit (admission) odds ratio (*p* = 0, 0.218) and 0.024 times decreased creatinine (admission) odds ratio (*p* = 0.679).

**Figure 1:**
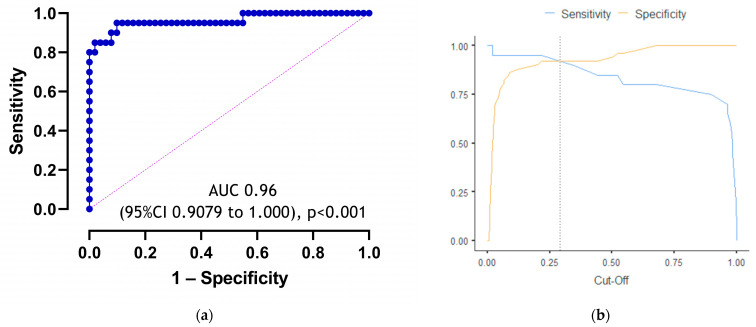
(**a**) ROC curve characterizing the dependence of the probability of the need for oxygen supply on the value of logistic function P. This ROC curve assesses the quality of logistic regression for predicting the primary outcome. It was created using the prediction results of the regression model and the category we are trying to predict. (**b**) Cut-off plot with the best cut-off point to maximize specificity and sensitivity indicators.

When evaluating the dependence of the probability of odds on the value of logistic function *p* using the ROC analysis, the following curve was obtained (Figure 1).

The area under the ROC curve comprised 0.96 with 95% CI: 0.91–1.00. The resulting model was statistically significant (*p* < 0.001).

The cut-off value of the logistic function *p* which corresponds to the highest Youden’s J statistic is 0.29. The specificity and sensitivity of the method were 92.2% and 90%, respectively (Figure 1).

### 3.2. Clinical and Laboratory Findings in Patients Treated with Pavloxid vs. Standard Therapy

At admission, out of 72 patients, Paxlovid was prescribed to 23 patients (11 patients with MAFLD and 12 patients without MAFLD). Patients were divided into four groups (27 COVID-19 patients with standard treatment, 12 COVID-19 patients with Paxlovid treatment, 22 COVID-19 with MAFLD patients with standard treatment, and 11 COVID-19 + MAFLD patients with Paxlovid treatment).

Next, we assessed the effect of Paxlovid on the main outcomes among the entire cohort (patients treated with Paxlovid vs. standard treatment, patients with existing MAFLD vs. non-MAFLD and between four groups (COVID-19—standard treatment, COVID-19—Paxlovid treatment, COVID-19 with MAFLD—standard treatment and COVID-19 with MAFLD—Paxlovid treatment).

**Figure 2:**
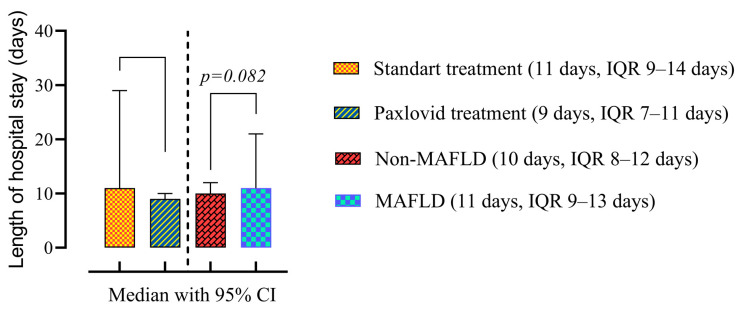
Comparison of the medians of four groups: patients treated with standard therapy vs. those treated with Paxlovid (disregarding the presence of MALFD) on the left; patients with and without MAFLD (disregarding the treatment) on the right. Data are presented as medians and *p*-values were calculated using the Mann–Whitney test. IQR—25–75% interquartile range.

Paxlovid-treated patients had significantly lower lengths of hospital stay (9 days, IQR 7–11 days vs. 11 days, IQR 9–14 days, *p* = 0.001). The presence of MAFLD itself did not affect the duration of hospitalization (Figure 2).

**Figure 3:**
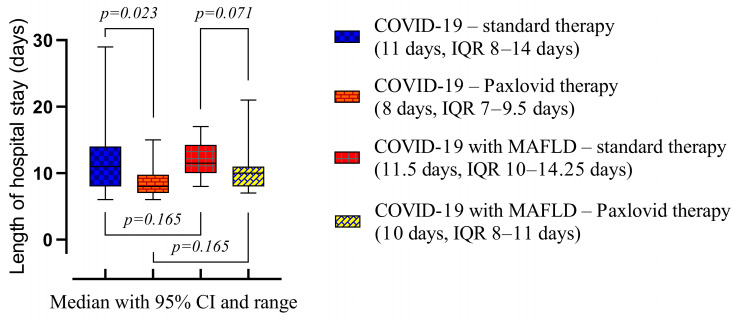
Comparison of the medians of four groups (COVID-19—standard treatment, COVID-19—Paxlovid treatment, COVID-19 with MAFLD—standard treatment, and COVID-19 with MAFLD—Paxlovid treatment) during hospitalization. Data are presented as medians and *p*-values were calculated using the Mann–Whitney test. IQR—25–75% interquartile range.

Paxlovid treatment significantly reduced the length of hospital stay in both COVID-19 with MAFLD (10 days, IQR 8–11 days vs. 11.5 days, IQR 10–14.25 days, *p* = 0.025) and COVID-19 without MAFLD (8 days, IQR 7–9 days vs. 11 days, IQR 8–14 days, *p* = 0.018) cohort (Figure 3). The presence of MALFD did not show any significant effect on the duration of hospitalization in both Paxlovid and standard treatment cohorts.

**Figure 4:**
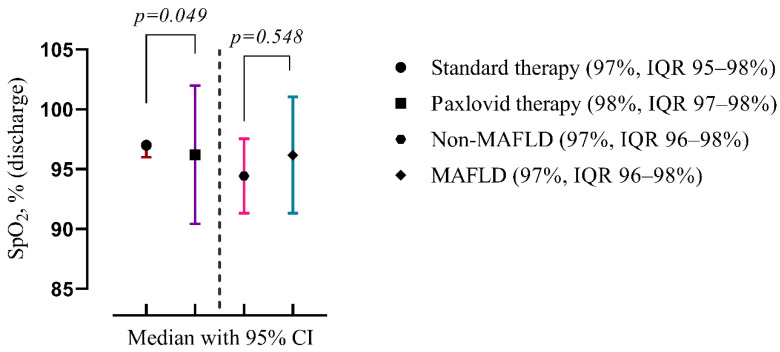
Comparison of the medians of four groups: patients treated with standard therapy vs. those treated with Paxlovid (disregarding the presence of MALFD) on the left; patients with and without MAFLD (disregarding the treatment) on the right. Data are presented as medians and *p*-values were calculated using the Mann–Whitney test. IQR—25–75% interquartile range.

On admission patients treated with Paxlovid had significantly higher oxygen saturation levels (98%, IQR 97–98% vs. 97%, IQR 95–98%, *p* = 0.049). The presence of MAFLD did not affect the blood saturation level (Figure 4).

**Figure 5:**
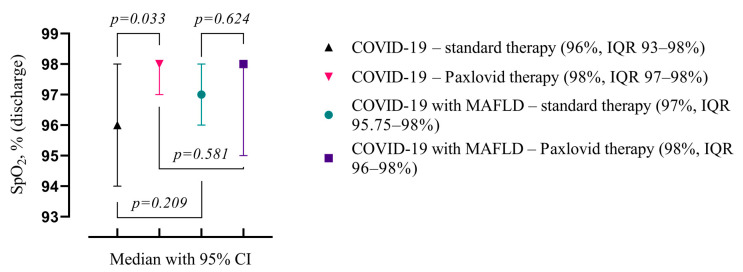
Comparison of the medians of four groups (COVID-19—standard treatment, COVID-19—Paxlovid treatment, COVID-19 with MAFLD—standard treatment, and COVID-19 with MAFLD—Paxlovid treatment) during hospitalization. Data are presented as medians and *p*-values were calculated using the Mann–Whitney test. IQR—25–75% interquartile range.

Comparing patients with COVID-19, Paxlovid treatment significantly connected with higher blood oxygen saturation levels on discharge in the non-MAFLD cohort (98%, IQR 97–98% vs. 96%, IQR 93–98%, *p* = 0.033) but not in MAFLD cohort. MAFLD itself did not influence the blood oxygenation (Figure 5).

**Figure 6:**
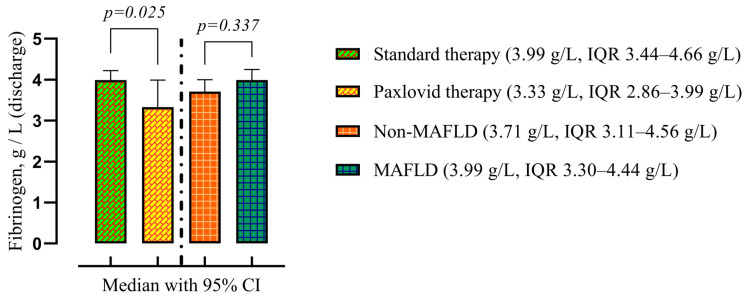
Comparison of the medians of four groups: patients treated with standard therapy vs. those treated with Paxlovid (disregarding the presence of MALFD) on the left; patients with and without MAFLD (disregarding the treatment) on the right. Data are presented as medians and *p*-values were calculated using the Mann–Whitney test. IQR—25–75% interquartile range.

Additionally, the Paxlovid treatment group had a lower fibrinogen level (3.33 g/L, IQR 2.86–3.99 g/L vs. 3.99 g/L, IQR 3.44–4.66 g/L, *p* = 0.025) on discharge. The presence of MAFLD did not affect the fibrinogen level (Figure 6).

**Figure 7:**
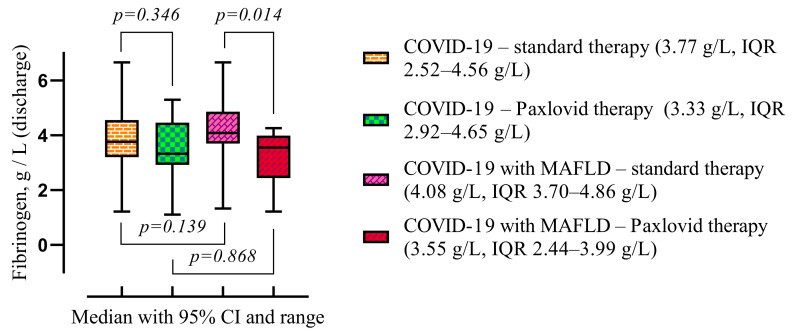
Comparison of the medians of four groups (COVID-19—standard treatment, COVID-19—Paxlovid treatment, COVID-19 with MAFLD—standard treatment, and COVID-19 with MAFLD—Paxlovid treatment) during hospitalization. Data are presented as medians and *p*-values were calculated using the Mann–Whitney test. IQR—25–75% interquartile range.

The only statistically significant difference in fibrinogen levels on discharge was observed between the COVID-19 with MAFLD groups. Fibrinogen level was lower after Paxlovid therapy (3.55 g/L, IQR 2.44–3.99 vs. 4.08 g/L, IQR 3.70–4.86, *p* = 0.014) compared with standard therapy (Figure 7).

**Figure 8:**
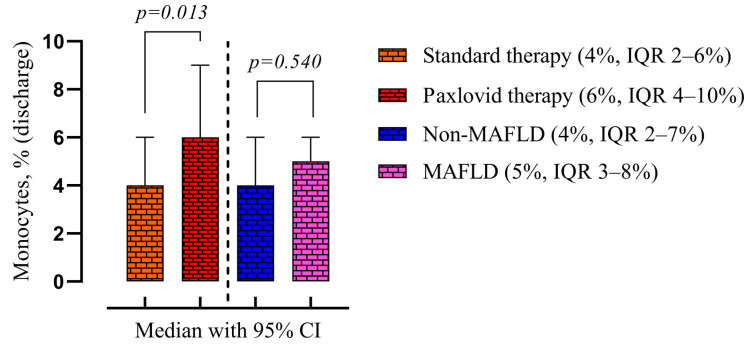
Comparison of the medians of four groups: patients treated with standard therapy vs. those treated with Paxlovid (disregarding the presence of MALFD) on the left; patients with and without MAFLD (disregarding the treatment) on the right. Data are presented as medians and *p*-values were calculated using the Mann–Whitney test. IQR—25–75% interquartile range.

There was also observed a higher monocyte level in the Paxlovid-treated group (6%, IQR 4–10% vs. 4%, IQR 2–6%, *p* = 0.013) compared with standard treatment. MAFLD cohort also had a higher monocyte level but was not statistically significant (Figure 8).

**Figure 9:**
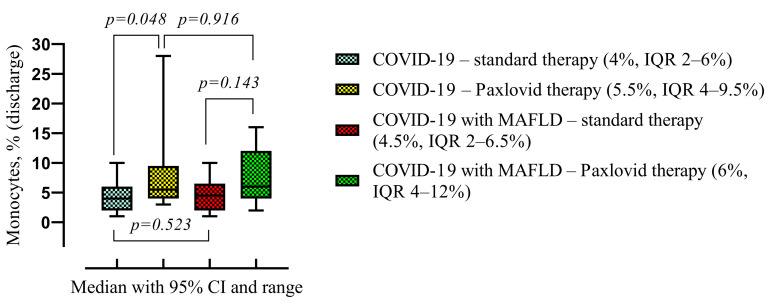
Comparison of the medians of four groups (COVID-19—standard treatment, COVID-19—Paxlovid treatment, COVID-19 with MAFLD—standard treatment, and COVID-19 with MAFLD—Paxlovid treatment) during hospitalization. Data are presented as medians and *p*-values were calculated using the Mann–Whitney test. IQR—25–75% interquartile range.

We observed a statistically significant difference in COVID-19 non-MAFLD cohort. Patients treated with Paxlovid had a higher monocyte level (5.5%, IQR 4–9.5% vs. 4%, IQR 2–6%, *p* = 0.048) compared with standard therapy (Figure 9).

### 3.3. Difference in Laboratory Findings in Patients Treated with Paxlovid and Standard Therapy on Discharge Comparing with Admission

**Figure 10:**
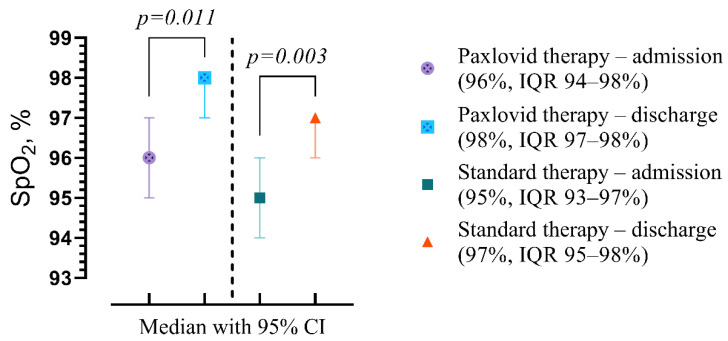
The difference in the medians of the clinical and laboratory findings in patients with Paxlovid and standard therapy at discharge compared with admission. Data are presented as medians with IQR, and *p*-values were calculated using Wilcoxon matched-pairs test. IQR—5–75% interquartile range.

We also examined the impact of Paxlovid and standard therapy on laboratory findings at the end of treatment compared with admission. Both groups Paxlovid (96%, IQR 94–98% vs. 98%, IQR 97–98%, *p* = 0.011) and standard treatment (95%, IQR 93–97% vs. 97%, IQR 95–98%, *p* = 0.003) demonstrated a statistically significant difference with increased SpO_2_ level on discharge (Figure 10).

**Figure 11:**
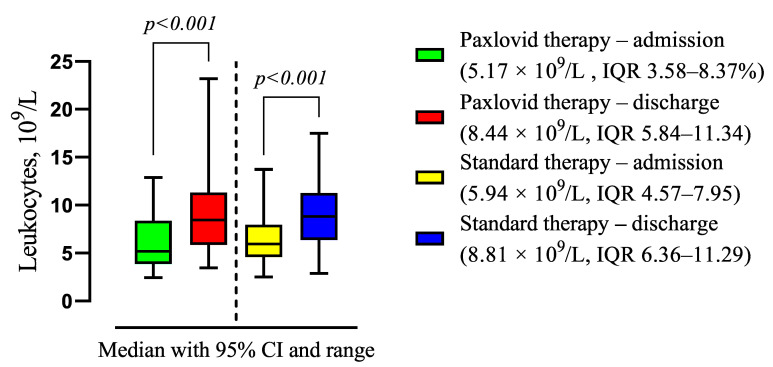
The difference in the medians of the clinical and laboratory findings in patients with Paxlovid and standard therapy at discharge compared with admission. Data are presented as medians with IQR, and *p*-values were calculated using Wilcoxon matched-pairs test. IQR—25–75% interquartile range.

There was revealed statistically significant increased leukocyte level on discharge for Paxlovid (5.17 × 10^9^/L, IQR 3.58–8.37% vs. 8.44 × 10^9^/L, IQR 5.84–11.34, *p* < 0.001) and standard treatment (5.94 × 10^9^/L, IQR 4.57–7.95 vs. 8.81 × 10^9^/L, IQR 6.36–11.29, *p* < 0.001) group (Figure 11).

**Figure 12:**
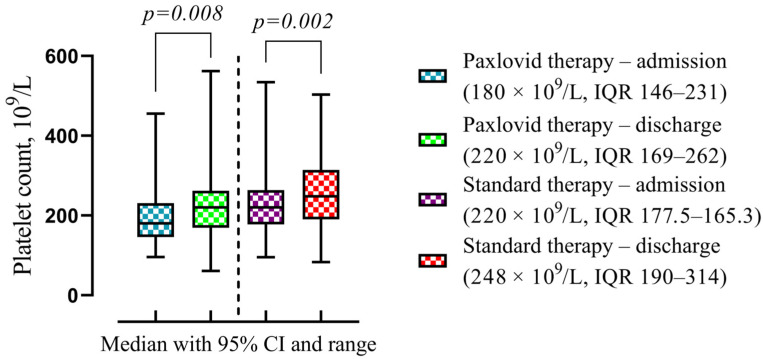
The difference in the medians of the clinical and laboratory findings in patients with Paxlovid and standard therapy at discharge compared with admission. Data are presented as medians with IQR, and *p*-values were calculated using Wilcoxon matched-pairs test. IQR—25–75% interquartile range.

It was found to be a statistically significant increase in platelet count level on discharge for Paxlovid (180 × 10^9^/L, IQR 146–231 vs. 220 × 10^9^/L, IQR 169–262, *p* = 0.008) and standard treatment (220 × 10^9^/L, IQR 177.5–165.3 vs. 248 × 10^9^/L, IQR 190–314, *p* = 0.002) group (Figure 12).

Band neutrophil levels were statistically significantly lower in both Paxlovid (7%, IQR 6–12 vs. 3%, IQR 2–4, *p* < 0.001) and standard therapy (9%, IQR 5.5–14.5 vs. 3%, IQR 2–5.5, *p* < 0.001) group on discharge comparing with admission. Difference between findings on discharge was not significant. APTT level decreased on discharge (33.4 s, IQR 29.8–37 vs. 29.8 s, IQR 25.6–34.7, *p* = 0.014)—Paxlovid group and (33.7 s, IQR 30.6–36.25 vs. 31 s, IQR 26.9–34.15, *p* < 0.001) standard therapy group. AST was higher in the Paxlovid group (22.5, IQR 17.4–25.8 vs. 30.8, IQR 24.5–76.9, *p* = 0.003) and standard therapy group (25.1, IQR 17.55–33.45 vs. 35, IQR 24.75–61, *p* < 0.001). Other clinical findings are shown in Table 5.

### 3.4. Correlation Analysis

**Figure 13:**
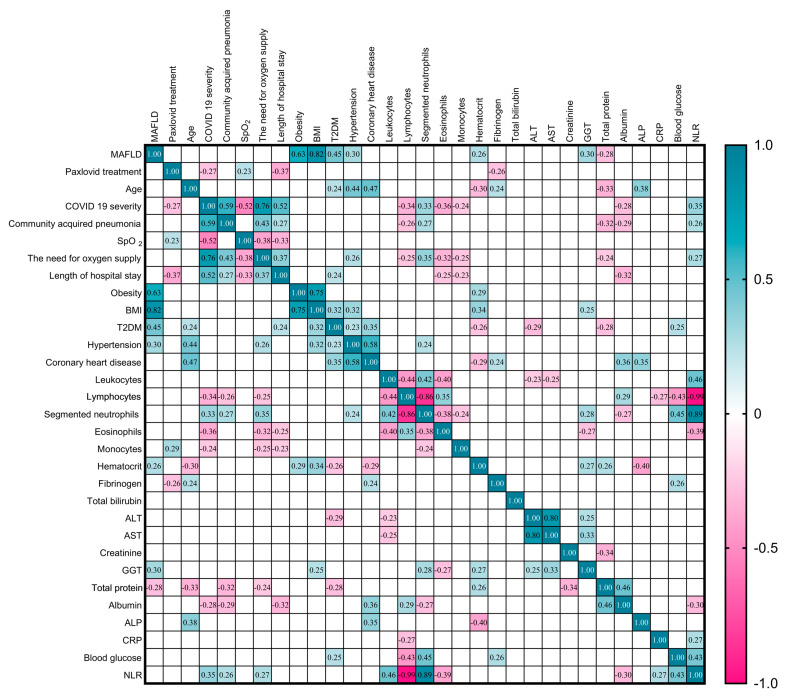
Correlation correlogram. Spearman’s correlation was used with two continuous variables, point-biserial correlation between binary and continuous data, the Chi-square test between two binary data. The color at the intersection of those variables represents the strength of the correlation between two variables. Colors range from crimson (strong negative correlation; r = −1.0) to cyan blue (strong positive correlation; r = 1.0). Results were not represented if *p* > 0.05.

Next, we analyzed potential correlations between baseline patients’ characteristics, and clinical and laboratory findings in patients with COVID-19, as presented in Figure 13. The presence of MAFLD correlated positively with BMI (r = 0.82, *p* < 0.001), obesity (r = 0.63, *p* < 0.001), T2DM (r = 0.42, *p* < 0.001), presence of hypertension (r = 0.30, *p* = 0.010), hematocrit (r = 0.26, *p* = 0.027), GGT (r = 0.30, *p* = 0.011) and negatively correlated with total protein (r = −0.28, *p* = 0.018). Paxlovid treatment showed negative correlation with COVID-19 severity (r = −0.28, *p* = 0.023), length of hospital stay (r = −0.37, *p* = 0.001), fibrinogen (r = −0.26, *p* = 0.025) and positive correlation with SpO_2_ (r = 0.23, *p* = 0.011). COVID-19 severity correlated positively with the need for oxygen supply (r = 0.76, *p* < 0.001), community-acquired pneumonia (r = 0.59, *p* < 0.001), length of hospital stay (r = 0.52, *p* < 0.001), segmented neutrophils (r = 0.33, *p* = 0.005), and NLR (r = 0.35, *p* = 0.003), and correlated negatively with SpO_2_ (r = −0.52, *p* < 0.001), lymphocytes (r = −0.34, *p* = 0.003), eosinophils (r = −0.36, *p* = 0.002), monocytes (r = −0.24, *p* = 0.040), and albumin (r = −0.28, *p* = 0.017). Length of hospital stay had negative correlation with SpO_2_ (r = 0.33, *p* = 0.005), eosinophils (r = −0.25, *p* = 0.035), monocytes (r = −0.23, *p* = 0.048), albumin (r = −0.32, *p* = 0.006), and positive correlation with T2DM (r = 0.24, *p* < 0.045), the need for oxygen supply (r = 0.43, *p* = 0.002) and community-acquired pneumonia (r = 0.27, *p* = 0.020).

Other strong and moderate positive correlations were between NLR with segmented neutrophils (r = 0.89, *p* < 0.001) and blood glucose (r = 0.43, *p* < 0.001); BMI and obesity (r = 0.75, *p* < 0.001); coronary heart disease with hypertension (r = 0.58, *p* < 0.001) and age (r = 0.44, *p* < 0.001); community-acquired pneumonia and the need for oxygen supply (r = 0.43, *p* < 0.001); and leukocytes with segmented neutrophils (r = 0.42, *p* = 0.005) and NLR (r = 0.46, *p* = 0.005). The rest of the strong and moderate negative correlations were between lymphocytes with segmented neutrophils (r = −0.86, *p* < 0.001), NLR (r = −0.99, *p* < 0.001), and blood glucose (r = −0.43, *p* < 0.001); leukocytes with lymphocytes (r = −0.44, *p* < 0.001) and eosinophils (r = −0.40, *p* < 0.001); and hematocrit and ALP (r = −0.40, *p* = 0.001).

### 3.5. Kaplan–Meier Test for Recovery Time

**Figure 14:**
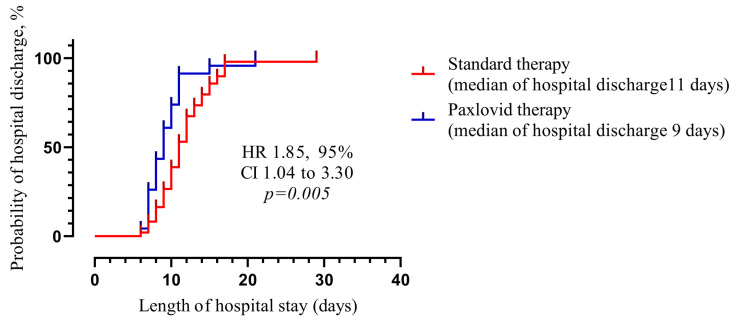
Association of time to recovery with Paxlovid prescription using Kaplan–Meier curves in patients with COVID-19. Hazard ratios (HR) with 95% confidence intervals and *p*-values were calculated using the log-rank test. We defined the probability of hospital discharge in a given length of time while considering time in many small intervals. The day of discharge from the hospital was considered the target event. *p* > 0.05 shows statistically significant difference between medians of hospital discharge (standard therapy—11 days vs. Paxlovid therapy—9 days).

The Kaplan–Meier involves computing probabilities of the occurrence of an event at a certain point in time. We examined the impact of Paxlovid treatment on time to recovery, as defined by time to hospital discharge. In survival analysis using Kaplan–Meier estimates, the appointment of the Paxlovid (HR 1.85, 95% CI 1.04 to 3.30, *p* = 0.005) appeared to be an efficient prognostic marker associated with shorter time to recovery, as presented in Figure 14.

We create a simple logistic regression for predicting Paxlovid therapy (Table 6). This predictive model has developed conditioning on SpO_2_ (admission), length of hospital stay (days), monocytes (discharge), and fibrinogen (discharge).

The resultant regression model exhibits statistical significance (*p* < 0.001). A Nagelkerke R^2^ value of 0.321 suggests a robust relationship between predictors and Paxlovid treatment. This model achieves an accuracy of 68.1%, accurately classifying the predictions.

The current model showed 0.034 times decreased SpO_2_ (discharge) odds ratio (*p* = 0.459), 0.238 times decreased the length of hospital stay odds ratio (*p* = 0.038), 0.207 times increased monocytes (discharge) odds ratio (*p* = 0.022), and 0.533 times decreased fibrinogen (discharge) odds ratio (*p* = 0.034).

**Figure 15:**
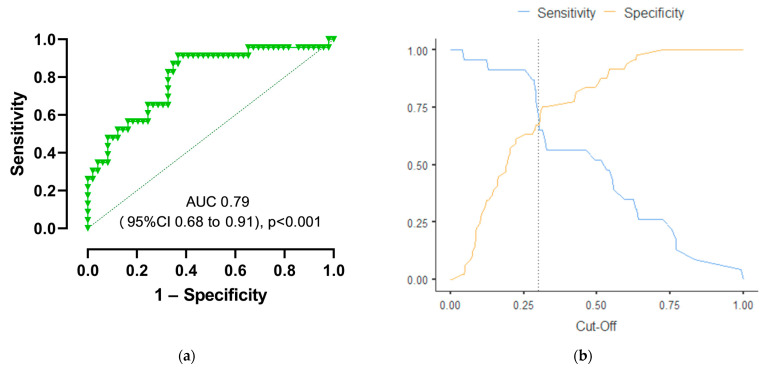
(**a**) ROC curve characterizing the dependence of the probability of the need for oxygen supply on value of logistic function P. This ROC curve assesses the quality of logistic regression for predicting the primary outcome. It was created using the prediction results of the regression model and the category we are trying to predict; (**b**) cut-off plot with the best cut-off point to maximize specificity and sensitivity indicators.

When evaluating the dependence of the probability of odds on the value of logistic function *p* using the ROC analysis, the following curve was obtained (Figure 15).

The area under the ROC curve comprised 0.79 with 95% CI: 0.68–0.91. The resulting model was statistically significant (*p* < 0.001).

The cut-off value of logistic function *p* which corresponds to the highest Youden’s J statistic is 0.3. The specificity and sensitivity of the method were 67.3% and 69.6%, respectively.

## 4. Discussion

In this investigation, we aimed to assess the efficacy of Paxlovid (nirmatrelvir/ritonavir) in individuals with COVID-19, specifically considering the coexistence of MAFLD. No significant distinctions were observed between the MAFLD and non-MAFLD cohorts in terms of hospitalization duration, blood oxygen saturation, and oxygen supplementation requirements. Notably, Paxlovid treatment correlated with a reduction in hospitalization duration and elevated oxygen saturation levels at discharge, irrespective of the presence or absence of MAFLD.

Furthermore, no significant correlation was established between the severity of COVID-19 and the presence of MAFLD. However, a noteworthy association was identified between the severity of COVID-19, the occurrence of community-acquired pneumonia, diminished oxygen saturation levels, and the necessity for oxygen support.

It is imperative to acknowledge that these findings are applicable solely to the specified patient cohort, as the study was exclusively conducted among individuals of European origin (Ukrainians) aged 20 to 70 years. The observed results consider the presence of the aforementioned concurrent diseases and additional characteristics outlined in Table 1.

MAFLD manifests in approximately one in every four individuals globally, establishing it as one of the most prevalent causes of chronic liver disease (CLD) [25]. Extant research has established a correlation between MAFLD and the manifestation of severe COVID-19 [26,27]. Notably, individuals with MAFLD exhibit an elevated likelihood of experiencing abnormal liver function, thus heightening their susceptibility to the progression of COVID-19 [28]. The risk of developing severe COVID-19 is more than twofold higher among MAFLD patients compared to those without MAFLD, particularly for individuals below the age of 60 [29]. Furthermore, there exists a recurrent association between patients with both metabolic syndrome (MetS) and abnormal liver function, leading to an increased incidence of Intensive Care Unit (ICU) admissions and a more severe trajectory of COVID-19 [30,31,32,33].

Hence, existing literature posits that individuals with MAFLD may be at augmented risk of experiencing severe COVID-19 [1,11,34,35,36], necessitating intensive care and supervision, requiring ICU-level supervision and care [1,34]. Nevertheless, the body of evidence on this association is not devoid of conflicting data. Notably, a meta-analysis conducted by Li et al. in 2022 [37] failed to identify conclusive evidence supporting MAFLD as an independent risk factor for severe COVID-19. Instead, the study suggested that the apparent connection between MAFLD and COVID-19 severity may be explicable by the concurrent presence of obesity within this patient cohort. This assumption is explained by immune dysregulation observed in individuals with elevated BMI, thereby exacerbating COVID-19 symptoms. When considered collectively, effective weight control emerges as a potentially pivotal modifiable risk factor for averting the progression to severe COVID-19 [37]. T2DM can also influence the immune system, potentially affecting the host response to COVID-19 [38]. The interaction between T2DM, MAFLD, and the immune response to COVID-19 may lead to nuanced and interconnected effects that are difficult to disentangle [14].

The cellular entry of SARS-CoV-2 is facilitated through binding to angiotensin-converting enzyme-2 (ACE-2) receptors in human cells [39,40]. This interaction is augmented by the fusion of the viral membrane with the host cell membrane, a process further facilitated by the priming of SARS-CoV-2 spike proteins through the activity of the host cell transmembrane protein, type II transmembrane serine protease (TMPRSS2) [39]. Notably, individuals with pre-existing MAFLD exhibit an elevated expression of ACE-2 receptors, thereby heightening their susceptibility to the development of severe COVID-19 disease [41]. Furthermore, observations by Shao et al. [42] revealed a noteworthy increase in the population of TMPRSS2+ cells in cirrhotic livers, thereby exacerbating COVID-19 outcomes. This study posited that pre-existing MAFLD might enhance susceptibility to the SARS-CoV-2 virus, primarily due to an elevated count of TMPRSS2+ progenitor cells.

MAFLD instigates a persistent low-grade inflammatory state, primarily mediated through insulin resistance, and is closely associated with obesity and DM [43]. These comorbidities, recognized contributors to adverse outcomes in COVID-19, are implicated in the chronic inflammatory milieu that detrimentally affects the immune system’s responsiveness to infections, potentially exacerbating the severity of COVID-19 infection [28,29]. The presence of pre-existing MAFLD further intensifies the acute inflammatory response induced by SARS-CoV-2 during active COVID-19 infection, leading to an escalation in the release of proinflammatory cytokines and reactive oxygen species [44,45].

In an investigation by Targher et al. [46], the relationship between imaging-defined MAFLD and the neutrophil-to-lymphocyte ratio (NLR) in MAFLD patients was scrutinized. The study revealed an elevated NLR and T lymphopenia in individuals with MAFLD compared to those without. Moreover, patients exhibiting increased NLRs experienced more adverse hospital outcomes, likely attributable to an augmented release of proinflammatory cytokines exacerbating the inflammatory/cytokine storm during active infection [46,47].

A retrospective analysis encompassing 202 individuals diagnosed with MAFLD revealed that these patients exhibited a prolonged period of viral shedding, lasting for 17.5 days in contrast to patients without MAFLD, who manifested a viral shedding duration of 12.1 days [28]. The protracted viral shedding in MAFLD patients is attributed to a compromised immune response and systemic inflammation, impeding effective containment of the virus within the host body. Additionally, the obese microenvironment in metabolic syndrome/MAFLD is posited to suppress interferon production and elevate ACE-2 receptor expression in COVID-19 infection, thereby exacerbating viral RNA replication. Consequently, these factors collectively contribute to heightened viral infectivity and increased severity of the infection [48]. These hypotheses underscore the synergistic nature of MAFLD and COVID-19 pathogenesis.

Numerous studies have presented evidence elucidating the reciprocal impact of liver diseases and COVID-19 on each other’s disease trajectory. Existing hepatic steatosis and MAFLD have been identified as influencers of COVID-19 disease severity, Intensive Care Unit (ICU) admission rates, and the necessity for invasive mechanical ventilation. Conversely, COVID-19 contributes to the exacerbation of hepatic injury and the progression of disease severity in MAFLD and other liver disorders [14]. However, it is essential to acknowledge that MAFLD frequently coexists with additional entities such as obesity and DM within the broader spectrum of metabolic syndrome. The intricate interplay between MAFLD and comorbidities like obesity and DM introduces challenges in establishing a direct causal link between MAFLD and COVID-19 outcomes independent of these associated comorbidities.

An avenue to comprehend the intricate interplay between MAFLD and COVID-19 involves the exploration of key genes and pathways implicated in these conditions. This approach holds promise for discerning potential drug targets and biomarkers. In a study by Karami et al. [49], a methodological framework encompassing weighted gene co-expression network analysis and LIME, an explainable artificial intelligence algorithm, was applied. This methodology successfully identified 17 novel FDA-approved candidate drugs. These drugs have the potential to be utilized in the treatment of COVID-19 patients through the regulation of four hub genes within the co-expression network. The identification of co-regulated gene networks and hub genes through such an approach has the capacity to unveil critical biological pathways.

Numerous genetic polymorphisms, such as PNPLA3 (rs738409), GCKR (rs780094), TM6SF2 (rs58542926), and LYPLAL1 (rs12137855), have undergone scrutiny concerning their association with MAFLD susceptibility and progression. Certain studies propose a plausible correlation between these MAFLD-associated polymorphisms and the severity of COVID-19 [35]. It is imperative to explore the potential synergistic effects of these genetic polymorphisms, thereby contributing to a comprehensive understanding of the intricate interplay between MAFLD susceptibility and the outcomes of COVID-19.

Individuals deemed at risk for developing severe and critical illness subsequent to COVID-19 infection are advised to undergo nirmatrelvir/ritonavir therapy [50]. Presently, the “Diagnosis and Treatment Protocol for Novel Coronavirus Pneumonia (Trial Version 9)” [50] advocates for the administration of this therapeutic regimen to COVID-19 patients within the initial 5 days following the onset of symptoms, with the aim of forestalling the progression to severe illness. Furthermore, the U.S. Food and Drug Administration (FDA) has granted approval for the use of this drug in adolescent patients aged 12 years and above, with a body weight of ≥40 kg [51].

Nirmatrelvir, functioning as a peptidomimetic inhibitor, specifically targets the main protease (Mpro) of the coronavirus, thereby impeding viral replication. Its primary metabolic pathway involves CYP3A4. Concurrently, ritonavir, an inhibitor of HIV-1 protease, enhances the blood concentration of nirmatrelvir by inhibiting the enzymatic activity of CYP3A4, thereby synergistically augmenting its effectiveness. The elimination pathways for these compounds differ, with nirmatrelvir primarily undergoing renal excretion and ritonavir undergoing hepatic metabolism [52].

A multitude of studies, encompassing vaccinated participants, consistently reported the efficacy of nirmatrelvir/ritonavir in reducing hospitalization and mortality rates, even in the context of prevalent omicron and BA4/5 variants. However, the observed degree of effectiveness exhibited variability across the spectrum of studies [53,54,55,56,57,58,59]. Several of these studies were conducted during the periods characterized by the Delta and Omicron variants, potentially leading to varying effectiveness compared to earlier stages. Nirmatrelvir/ritonavir exhibited favorable tolerance and efficacy in patients with the Omicron variant of COVID-19 [60].

In the EPIC-HR trial, among non-hospitalized individuals with mild-to-moderate COVID-19 who were unvaccinated and at risk of progressing to severe disease, the early initiation of nirmatrelvir plus ritonavir within 5 days of symptom onset resulted in a notable relative reduction of 88% in the composite outcome of hospitalization or death [18]. Conversely, the updated analysis of the EPIC-SR trial, which involved unvaccinated adults at standard risk of COVID-19 or fully vaccinated individuals with at least one risk factor, indicated a non-significant reduction of 51% in hospitalization or death with the use of nirmatrelvir plus ritonavir in non-hospitalized patients [61].

Nirmatrelvir–ritonavir treatment demonstrated an association with fewer emergency department visits in the 28 days following administration compared to matched, untreated patients. This finding aligns with a single-arm study by Malden and colleagues, which reported emergency department visits or hospitalizations occurring with less than 1% frequency in the 5–15 days after nirmatrelvir–ritonavir treatment [62]. Aggarwal NR, et al. [56] outed potential benefits of nirmatrelvir–ritonavir in both older and younger patients, as did Zhou X, et al. [63] and Shah M, et al. [64]. Notably, a study by Arbel and colleagues found a reduction in hospitalization only in COVID-19-positive outpatients aged 65 years or older after nirmatrelvir–ritonavir treatment, with no apparent benefit observed in those younger than 65 years [53].

The findings from the meta-analysis conducted by Amani B. et al. [65] underscored a significant association between Paxlovid treatment and a markedly lower mortality rate in COVID-19 patients compared to control groups. Notably, Paxlovid-treated individuals exhibited a significantly lower rate of hospitalization or death in comparison to those not receiving Paxlovid. These results align with the meta-analysis by Zheng et al. [66], who similarly demonstrated a reduction in the death rate among COVID-19 patients treated with Paxlovid, emphasizing a significant clinical benefit in terms of reduced hospitalization rates compared to those who did not receive Paxlovid. Furthermore, a meta-analysis encompassing three new oral antivirals—molnupiravir, fluvoxamine, and Paxlovid—revealed that Paxlovid treatment was linked to a significantly lower mortality rate in COVID-19 patients compared to placebo, highlighting the efficacy of Paxlovid, molnupiravir, and fluvoxamine in mitigating the hospitalization rate due to COVID-19 [67].

Results from a recently published randomized controlled trial (RCT) involving nonhospitalized adults at high risk of progression to COVID-19 [18] demonstrated a lower frequency of Grade 3 or 4 adverse events, serious adverse events, and adverse events leading to discontinuation in the Paxlovid group as opposed to the placebo group. Moreover, data from a large cohort of 183,041 COVID-19 patients indicated no significant difference between the Paxlovid and no antiviral treatments concerning a higher risk of abnormal liver enzymes or drug-induced liver injury (DILI) [68]. These findings are consistent with a meta-analysis examining adverse events associated with the oral antiviral molnupiravir, which showed no significant difference in the incidence of adverse events in COVID-19 patients compared to the control group [69].

Limited information is available regarding adverse events linked to nirmatrelvir/ritonavir, with recognized common occurrences encompassing dysgeusia and diarrhea [18,70,71]. The investigation conducted by Li et al. [72] examined prevalent adverse events linked to the administration of nirmatrelvir/ritonavir. Predominantly, these consequences were non-serious, with dysgeusia (17.55%), diarrhea (8.80%), nausea (5.31%), headache (4.77%), pyrexia (2.99%), vomiting (2.88%), and malaise (2.76%) being the most frequently reported. The findings indicated a significant association between the use of nirmatrelvir/ritonavir and the recurrence of COVID-19 [72].

The studies in the discussion section were heterogeneous in terms of study designs, patient populations, treatment protocols, the presence of randomization, patients vaccinated with different COVID-19 vaccines, and the absence of vaccination. There were variations in the severity of the disease in outpatient and inpatient treatment settings. However, nirmatrelvir–ritonavir (Paxlovid) demonstrated high efficacy across all cases.

In our study, we relied on the use of targeted antiviral therapy for COVID-19, as it did not affect the course of MAFLD. We tried to find out the effectiveness of nirmatrelvir–ritonavir (Paxlovid) treatment in such patients.

Nevertheless, we studied a well-defined cohort of patients and reported the first data examining the effectiveness of nirmatrelvir–ritonavir (Paxlovid) treatment in patients with MAFLD and COVID-19. Longitudinal studies are needed to find out the significance of targeted antiviral therapy for COVID-19 in patients with components of metabolic syndrome and MAFLD.

## 5. Conclusions

This investigation provides crucial insights into the potential benefits of targeted antiviral therapy, specifically Paxlovid, in patients with MAFLD and COVID-19. Although no significant distinctions were observed in hospitalization duration, oxygen saturation, or severity based on MAFLD status, Paxlovid treatment correlated with reduced hospitalization duration and improved oxygen saturation at discharge, regardless of MAFLD presence.

## Figures and Tables

**Table 1:** Baseline patients’ characteristics.

	MAFLD (*n* = 33)	Non-MAFLD (*n* = 39)	*p*-Value ^a^
Age, median (IQR) ^b^	66 (50–72)	65 (41–72)	0.560
Male, No. (%)	21 (63.6%)	22 (54.4%)	0.632
BMI, kg/m²	30.8 (28.42–33.5)	24 (22.4–25.35)	<0.001
Peripheral oxygen saturation (SpO_2_), % on admission/	95 (92–96)	96 (94–97)	0.264
discharge	97 (96–98)	97 (96–98)	0.545
The duration of hospital stay, days	11 (9–13)	10 (8–12)	0.082
COVID-19 severity (moderate/severe/critical), *n*	20/10/3	22/15/2	0.670
The need for oxygen supply, *n* (%)	12 (36.6%)	8 (20.51%)	0.188
Comorbidities			
Diabetes mellitus	14 (42.4%)	2 (6.6%)	<0.001
Arterial hypertension	25 (75.7%)	18 (46.15%)	0.160
COPD	1 (3%)	3 (7.69%)	0.620
Obesity	18 (54.5%)	0	<0.001
Coronary heart disease	14 (42.4%)	13 (33.33%)	0.471
Community-acquired pneumonia	18 (54.55%)	22 (54.41%)	1.000
Mortality	1 (3.03%)	2 (5.12%)	1.000

^a^ Fisher exact, Chi-square or Mann–Whitney U test, as appropriate; ^b^ data are presented as medians (interquartile range). Abbreviations: IQR—interquartile range; COPD—chronic obstructive pulmonary disease.

**Table 2:** Laboratory finding on admission/discharge.

	Admission			Discharge		
	MAFLD (*n* = 33)	Non-MAFLD (*n* = 39)	*p*-Value ^a^	MAFLD (*n* = 33)	Non-MAFLD (*n* = 39)	*p*-Value ^a^
Leukocytes, 10^9^/L, median (IQR) ^b^	6.4 (4.47–9.37)	5.57 (4.04–7.57)	0.139	8.93 (6.87–11.33)	7.73 (5.17–10.8)	0.083
Lymphocytes, %	22 (9.5–29.5)	23 (14–33)	0.428	27 (16.5–34)	29 (17–38)	0.406
Band neutrophils, %	9 (5.5–16)	7 (6–12)	0.185	3 (2–5)	3 (2–4)	0.950
Segmented neutrophils, %	62 (49.5–71)	62 (52–75)	0.874	64 (56–75)	63 (52–72)	0.490
Eosinophils, %	1 (0–2)	1 (0–1)	0.797	1 (0–1)	1 (0–1)	0.279
Monocytes, %	4 (2–6.5)	4 (1–9)	0.793	5 (3–8)	4 (2–7)	0.535
ESR *, mm/h	6 (4–19.5)	6 (4–11)	0.537	5 (4–7)	5 (4–14)	0.271
Platelet count, 10^9^/L	214 (168–250)	210 (170–258)	0.576	218 (183–314)	244 (178–290)	0.709
Hematocrit, %	40 (33.64–48.50)	36.9 (32–40.8)	0.055	37.8 (31.97–45.95)	34.5 (31.29–38.95)	0.028
INR *, *n*	0.99 (0.95–1.05)	1.03 (0.95–1.12)	0.244	1.01 (0.95–1.07)	1.02 (0.92–1.1)	0.888
PT *, s	12.8 (12.2–13.55)	12.8 (12.2–14)	0.553	13.2 (12.35–13.4)	12.4 (11.6–13.8)	0.419
QPT *, %	94.1 (87.1–104)	92.6 (76.7–100)	0.345	96 (81.6–106.1)	95 (82–103.6)	0.923
aPTT *, s	33.4 (29.8–37.4)	33.8 (30.8–35.4)	0.991	29.8 (25.2–33.9)	31.8 (27.6–35.4)	0.171
Fibrinogen, g/L	3.99 (3.1–5.21)	3.77 (3.33–5.11)	0.941	3.99 (3.3–4.44)	3.71 (3.12–4.56)	0.333
Total bilirubin, mmol/L	12.9 (10.8–15.15)	12.3 (10.8–16)	0.852	11.3 (10.6–16.4)	12.7 (10.5–16)	0.964
ALT *, mmol/L	28.2 (21.1–33.65)	25 (21.2–30.5)	0.354	30.6 (23.15–37.45)	27.5 (22.9–44)	0.747
AST *, mmol/L	27 (21.4–43.6)	22.9 (16.6–27.7)	0.024	42 (72.05–23.9)	33 (24.5–53.5)	0.584
Creatinine, mmol/L	104 (91–117)	90 (77–104)	0.015	99 (86–115)	94 (79–113)	0.197
GGT *, U/L	60 (35.5–87)	36 (23–66)	0.017	67 (41–93.5)	43 (30–65)	0.012
Total protein, g/L	70.9 (64.6–76.25)	61.1 (61.2–70.4)	0.016	63.4 (59.3–67.8)	67.6 (61.9–72.2)	0.019
Albumin, g/L	47 (43–46)	44 (40–55)	0.068	42 (39–47)	43 (37–49)	0.861
ALP *, mmol/L	123 (99.5–149.5)	127 (103–167)	0.584	111 (94.5–133.5)	125 (100–150)	0.124
CRP *, mg/L	12 (6–24)	6 (6–12)	0.006	6 (6)	6 (6)	0.806
Blood glucose, mmol/L	7 (5.6–9.5)	5.9 (5.2–7.1)	0.012	5.6 (5.05–7)	5.2 (4.7–6.3)	0.178
NLR *	3.32 (2.09–8.96)	2.83 (1.73–6)	0.381	2.48 (1.76–4.79)	2.36 (1.47–4.65)	0.480

^a^ Mann–Whitney U test, as appropriate; ^b^ data are presented as medians (interquartile range). Abbreviations *: AST—Aspartate Aminotransferase; ALT—Alanine Aminotransferase; GGT—Gamma-glutamyl Transferase; INR—International Normalized Ratio; ESR—Erythrocyte Sedimentation Rate; CRP—C-reactive protein; NLR—the neutrophil-to-lymphocyte ratio; ALP—Alkaline Phosphatase; aPTT—Activated partial thromboplastin time; PT—Prothrombin Time; QPT—Quick prothrombin time. The Mann–Whitney U test was used to compare the two independent groups.

**Table 3:** Estimating parameters in multinomial logistic regression for COVID-19 severity.

COVID-19 Severity ^a^		B (OR ^b^)	Std. Error	Wald	df	Sig. (*p*-Value)	Exp (B)	95% CI for Exp (B)	
								Lower Bound	Lower Bound
Severe COVID-19 ^a^	Intercept	110.170	27.906	15.586	1	0.000			
	SpO_2_ (admission)	−1.123	0.280	16.125	1	0.000	0.325	0.188	0.563
	Lymphocytes, % (admission)	−0.051	0.036	1.974	1	0.160	0.950	0.885	1.020
	QPT, % (admission)	−0.004	0.025	0.022	1	0.882	0.996	0.948	1.047
	Albumin, g/L (admission)	−0.050	0.043	1.401	1	0.237	0.951	0.875	1.034
Critical COVID-19 ^a^	Intercept	236.719	132.155	3.208	1	0.073			
	SpO_2_ (admission)	−2.382	1.414	2.838	1	0.092	0.092	0.006	1.476
	Lymphocytes, % (admission)	0.079	0.232	0.117	1	0.733	1.082	0.687	1.706
	QPT, % (admission)	−0.206	0.234	0.778	1	0.378	0.814	0.515	1.287
	Albumin, g/L (admission)	−0.082	0.338	0.059	1	0.808	0.921	0.475	1.787

^a^ The reference category is moderate COVID-19 severity, ^b^ odds ratio.

**Table 4:** Estimating parameters in logistic regression for the need for oxygen supply.

	B (OR ^a^)			df	Sig. (*p*-Value)		95% CI for EXP (B)	
		S.E.	Wald			Exp (B)	Lower	Upper
SpO_2_ admission	−1.245	0.375	11.019	1	0.001	0.288	0.138	0.601
Leukocytes, 10^9^/L (admission)	0.261	0.215	1.469	1	0.225	1.298	0.851	1.980
Hematocrit (%) (admission)	0.067	0.054	1.518	1	0.218	1.069	0.961	1.188
Creatinine, mmol/L (admission)	−0.002	0.004	0.171	1	0.679	0.998	0.990	1.007
Constant	112.063	34.734	10.409	1	0.001	4.659 × 10^48^		

Variable(s) entered on step 1: SpO_2_ admission; leukocytes, 10^9^/L (admission); hematocrit, % (admission); creatinine, mmol/L (admission); ^a^ odds ratio.

**Table 5:** Difference in laboratory findings in patients treated with Paxlovid/standard therapy on discharge.

Admission/Discharge, *p*-Value	Clinical and Laboratory Findings	Paxlovid Therapy (*n* = 23)	Standard Therapy (*n* = 49)	*p*-Value
A	Peripheral oxygen saturation (SpO_2_), %	96 (94–98)	95 (93–97)	*p* = 0.187
D		98 (97–98)	97 (95–98)	*p* = 0.049
*p*		*p* = 0.011	*p* = 0.003	
A	Leukocytes, 10^9^/L	5.17 (3.85–8.37)	5.94 (4.57–7.95)	*p* = 0.567
D		8.44 (5.84–11.34)	6.36 (8.81–11.29)	*p* = 0.978
*p*		*p* < 0.001	*p* < 0.001	
A	Lymphocytes, %	25 (16–33)	22 (10–31)	*p* = 0.291
D		24 (17–37)	28 (17–34)	*p* = 0.566
*p*		*p* = 0.466	*p* = 0.002	
A	Band neutrophils, %	7 (6–12)	9 (5.5–14.5)	*p* = 0.720
D		3 (2–4)	3 (2–5.5)	*p* = 0.398
*p*		*p* < 0.001	*p* < 0.001	
A	Segmented neutrophils, %	59 (46–70)	63 (54–72.5)	*p* = 0.308
D		66 (52–75)	62 (54.5–70)	*p* = 0.283
*p*		*p* = 0.008	*p* = 0.758	
A	Eosinophils, %	1 (1–2)	1 (0–1)	*p* = 0.173
D		1 (0–1)	1 (0–1.5)	*p* = 0.631
*p*		*p* = 0.079	*p* = 0.647	
A	Monocytes, %	5 (2–9)	4 (1.5–6)	*p* = 0.072
D		6 (4–10)	4 (2–6)	*p* = 0.013
*p*		*p* = 0.626	*p* = 0.674	
A	ESR, mm/h	5 (4–11)	6 (4–19.5)	*p* = 0.432
D		5 (4–6)	5 (4–10)	*p* = 0.418
*p*		*p*= 0.094	*p* = 0.102	
A	Platelet count, 10^9^/L	180 (146–231)	220 (177.5–263.5)	*p* = 0.055
D		220 (169–262)	248 (190–314)	*p* = 0.257
*p*		*p* = 0.008	*p* = 0.002	
A	Hematocrit, %	37.3 (34.2–44)	38.11 (30.87–43.5)	*p* = 0.291
D		37 (32–42)	36 (31.43–42.97)	*p* = 0.511
*p*		*p* = 0.075	*p* = 0.115	
A	INR, *n*	0.98 (0.95–1.1)	1 (0.95–1.09)	*p* = 0.473
D		1.01 (0.88–1.06)	1.02 (0.93–1.09	*p* = 0.205
*p*		*p* = 0.955	*p* = 0.655	
A	PT, sec	12.7 (12.2–13.6)	12.9 (12.2–13.85)	*p* = 0.650
D		12.6 (11.9–13.4)	12.8 (11.85–13.65)	*p* = 0.522
*p*		*p* = 0.479	*p* = 0.484	
A	QPT, %	94.1 (85.3–105.2)	93.6 (82.6–101)	*p* = 0.437
D		96.2 (84.3–106.1)	91.8 (80.9–104)	*p* = 0.625
*p*		*p* = 0.949	*p* = 0.575	
A	APTT, s	33.4 (29.8–37)	33.7 (30.6–36.25)	*p* = 0.762
D		29.8 (25.6–34.7)	31 (26.9–34.15)	*p* = 0.547
*p*		*p* = 0.014	*p* < 0.001	
A	Fibrinogen, g/L	3.99 (3.55–4.88)	3.55 (3.1–5.32)	*p* = 0.454
D		3.33 (2.86–3.99)	3.99 (3.44–4.66)	*p* = 0.025
*p*		*p* = 0.018	*p* = 0.430	
A	Total bilirubin, mmol/L	12.9 (10.8–17.1)	12.6 (10.8–15.3)	*p* = 0.998
D		11.2 (10.6–14.1)	12.7 (10.55–16.7)	*p* = 0.239
*p*		*p* = 0.092	*p* = 0.638	
A	ALT, mmol/L	23.4 (18.2–32.6)	25.4 (22–31.1)	*p* = 0.283
D		29.8 (23.9–36.6)	29 (22–39.3)	*p* = 0.690
*p*		*p* = 0.153	*p* = 0.031	
A	AST, mmol/L	22.5 (17.4–25.8)	25.1 (17.55–33.45)	*p* = 0.232
D		30.8 (24.5–76.9)	35 (24.75–61)	*p* = 1.000
*p*		*p* = 0.003	*p* < 0.001	
A	Creatinine, mmol/L	101 (80–117)	98 (81.5–113)	*p* = 0.950
D		96 (84–103)	96 (80–117.5)	*p* = 0.451
*p*		*p* = 0.325	*p* = 0.524	
A	GGT , unit/L	54 (26–83)	49 (28–75.5)	*p* = 0.978
D		52 (37–72)	57 (33–91.5)	*p* = 0.813
*p*		*p* = 0.310	*p* = 0.006	
A	Total protein, g/L	68.5 (61.6–74.6)	68.6 (62.65–72.1)	*p* = 0.959
D		66.3 (61.8–71.2)	63.6 (60.4–70.45)	*p* = 0.350
*p*		*p* = 0.345	*p* = 0.004	
A	Albumin, g/L	50 (45–56)	44 (40–52.5)	*p* = 0.040
D		40 (46–51)	42 (37.5–46)	*p* = 0.060
*p*		*p* = 0.102	*p* = 0.001	
A	ALP, mmol/L	138 (116–157)	119 (95–160.5)	*p* = 0.095
D		120 (96–146)	115 (99–148)	*p* = 0.785
*p*		*p* = 0.064	*p* = 0.284	
A	CRP, mg/L	6 (6–12)	6 (6–24)	*p* = 0.860
D		6 (6)	6 (6–12)	*p* = 0.104
*p*		***p* =** 0.008	*p* = 0.096	
A	Blood glucose, mmol/L	5.7 (5.1–7.5)	6.6 (5.5–8.05)	*p* = 0.239
D		5.6 (4.8–7.4)	5.5 (4.85–6.1)	*p* = 0.484
*p*		*p*= 0.325	*p* < 0.001	
A	NLR	2.83 (1.76–5)	3.32 (2–8.4)	*p* = 0.260
D		3.04 (1.42–5.12)	2.19 (1.68–4.62)	*p* = 0.547
*p*		*p*= 0.622	*p* = 0.009	

Wilcoxon signed-rank test was used for comparing two related groups. Mann–Whitney U test was used to compare the two independent groups.

**Table 6:** Estimating factors in logistic regression factors to predict Paxlovid therapy.

	B (OR ^a^)	Std. Error	Wald	df	Sig. (*p*-Value)	Exp (B)	95% C.I. for EXP (B)	
							Lower	Upper
SpO_2_ (discharge)	−0.034	0.046	0.548	1	0.459	0.967	0.884	1.057
Length of hospital stay (days)	−0.238	0.115	4.290	1	0.038	0.789	0.630	0.987
Monocytes, % (discharge)	0.207	0.091	5.229	1	0.022	1.230	1.030	1.469
Fibrinogen, g/L (discharge)	−0.533	0.251	4.494	1	0.034	0.587	0.359	0.961
Constant	5.726	5.094	1.263	1	0.261	306.735		

Variable(s) entered on step 1: SpO_2_ discharge; length of hospital stay (days); monocytes, % (discharge); fibrinogen, g/L (discharge); ^a^ odds ratio.

## Data Availability

Data are contained within the article.
